# Strategy to Promote the Biodegradation of Phenanthrene in Contaminated Soil by a Novel Bacterial Consortium in Slurry Bioreactors

**DOI:** 10.3390/ijerph19095515

**Published:** 2022-05-01

**Authors:** Xuyang Jiang, Zhen Mao, Licun Zhong, Jinbiao Yu, Yan Tang

**Affiliations:** School of Environmental Science and Spatial Informatics, China University of Mining and Technology, Xuzhou 221116, China; ts19160140p31@cumt.edu.cn (X.J.); ts20160201p31@cumt.edu.cn (L.Z.); ts20160180p31@cumt.edu.cn (J.Y.); ts20160082a31ld@cumt.edu.cn (Y.T.)

**Keywords:** microbial degradation, phenanthrene, extracellular polymeric substances, bioslurry

## Abstract

Polycyclic aromatic hydrocarbons (PAHs) are typical high-risk, persistent organic pollutants. Biological slurry reactors are widely used for enhanced bioremediation. In this experiment, a highly efficient phenanthrene-degrading bacteria group was obtained through screening and domestication, and the community was named MZJ_21. After the addition of MZJ_21 to the aerobic slurry bioreactor, with the optimum conditions of the temperature, stirring speed, and aeration rate of 30 °C, 120 rpm, and 1 L/min, respectively, the phenanthrene degradation ratio reached 95.41% within 48 h. The exploration of the degradation of phenanthrene by MZJ_21 indicated that most MZJ_21 communities adsorbed on the soil particle, mainly because MZI_21 could secrete extracellular polymers, which could stably adhere MZJ_21 on the solid phase. At the same time, the distribution ratio of phenanthrene in the solid phase is increased, so that the efficient phenanthrene degradation reaction takes place in the solid phase.

## 1. Introduction

Polycyclic aromatic hydrocarbons (PAHs) are typical high-risk persistent organic pollutants, which have received considerable attention due to their severe toxicities (carcinogenicity, teratogenicity, and mutagenicity) [1,2,3]. Moreover, PAHs have a high affinity towards soil organic matter and can remain stable in soil, thus posing a serious threat to natural ecosystems [4]. The sources of PAHs can be divided into natural sources and anthropogenic sources. The vast majority of PAHs in the environment come from human activities, such as energy production process and incomplete fuel combustion [5]. They usually exist in coal coking plant sites, fishing ports, and sewage irrigation farmland [6]. Since the end of the 20th century, many remediation methods of PAH pollution have achieved substantial progress [7]. According to their feature, they can be classified into thermal, physicochemical, chemical or biological treatments. Among them, bioremediation is one of the most common techniques used to treat PAH-contaminated soil and is a safe and efficient for soil remediation [8,9,10]. In the early years, this technology was widely used in large sites. Although there are other alternative technologies to repair large amounts of solid waste containing PAHs, the research on bioremediation has never stopped, and bioremediation is still considered to have great application prospects. According to the data of the 10th edition of the EPA Superfund remediation report, a total of 105 engineering projects used bioremediation technology from 1982 to 1999 [11]. For example, the French Limited Project in Texas uses a slurry bioreactor to repair the soil polluted by benzoapyrene. The treatment capacity of the reactor is 500 t/d, which reaches the repair goal in about 11 months, and the repair cost is about 169 dollars/t [12]. Southeastern wood preservation, a super fund project in Canton, Mississippi, uses a slurry bioreactor to mainly repair naphthalene, benzoapyrene, and other contaminated soil. The volume of the reactor is 130 m^3^, the treatment capacity is 50 t/d, the treatment time is 30~35 days, and the treatment cost is about 205 dollars/t [12]. Cai selected a typical oil contaminated site in Jianghan Oilfield to carry out the bioremediation demonstration project. By using the bioremediation technology combined with microorganisms and plants to treat the site, the degradation rate of petroleum hydrocarbons in surface soil reached 92.6–99.7% [13]. A slurry bioreactor, as an ectopic approach to bioremediation, presents a considerably improved reaction rate and removal efficiency of pollutants compared with in situ soil bioremediation [14,15,16,17]. In general, compared with other types of repair technologies, such as heat treatment and chemical treatment, although the construction and operation cost of slurry bioreactor is high, it will not cause secondary pollution in the repair process and reduce the secondary repair cost. Therefore, biological mud reactor is also economical.

Slurry bioreactors are excellent mixing containers, in which soil, water, microorganisms, and nutrients are completely mixed [18]. Slurry bioreactor presents the high degradation efficiency. Moreover, reaction conditions can be adjusted at any point of time; thus, bioreactors are always in the optimum operational state. The microbial activity is the key factor affecting the degradation efficiency of slurry bioreactors [7]. Therefore, adding efficient pollutant-degrading bacteria to slurry reactors effectively improves their degradation efficiency. However, most studies have focused on only the improvement of overall degradation performance of slurry reactors, and the local mechanism of pollutant removal has been ignored. Based on the previous research conclusions on the degradation mechanism, it is generally believed that: the main pollutant degradation mechanism in slurry bioreactors can be divided into three processes: solid–liquid mass transfer, gas–liquid mass transfer, and biodegradation. Generally, most microorganisms are more likely to degrade dissolved organic pollutants. Therefore, it is considered that most of the biodegradation processes are carried out in the liquid phase [19], while in the solid phase, biodegradation is possible only when microorganisms come into contact with organic pollution [20].

Extracellular polymeric substances (EPSs), which usually constitute polysaccharides, proteins, small amount of lipids, and nucleic acids [21], is a series of organic polymers secreted by microorganisms. When bacteria are in an unfavorable environment, bacterial cells will secrete more EPSs to protect themselves from toxic substances and extreme conditions [22]. Because there exhibit numerous negatively charged functional groups and their strong binding ability to organic pollutants, EPS could play an important role in the interfacial action. In many cases, EPS plays a similar role to surfactant, improving the hydrophobicity of organic matter. For example, EPS can desorb PAHs in soil through van der Waals force or emulsification, and improve the bioavailability of PAHs [23,24,25]. Therefore, EPS also plays an important role in the microbial degradation of PAHs [26,27].

In this paper, we domesticated aerobic-activated sludge with phenanthrene as the only source of carbon and energy and obtained a bacterial community (MZJ_21) that can efficiently degrade phenanthrene. By comparing the survival of MZJ_21 and natural soil communities in solid and liquid phases, as well as the distribution ratio of phenanthrene in solid and liquid phases in different experimental groups, combined with GC-MS, SEM, three-dimensional fluorescence, and high-throughput sequencing, the possible microbial degradation strategy in aerobic slurry bioreactor was revealed.

## 2. Materials and Methods

### 2.1. Chemicals

Phenanthrene (purity ≥ 97%) was purchased from CNW Technologies GmbH, Dusseldorf, Germany. Chromatographic grade ethyl acetate, chromatographic grade acetone, n-hexane, analytical grade methanol, and other analytical grade reagents were obtained from Sinopharm Chemical Reagent Co., Ltd., Shanghai, China.

### 2.2. Soil

The samples of natural soil used for experiments were collected from campus gardens in the China University of Mining and Technology (Xuzhou, Jiangsu, China). First, we removed the debris such as plants and stones in the soil, and then, we placed the soil in a shady place to air dry. After grinding and sieving through a 2-mm sieve, the soil was again air dried for 1 week before use. Contaminated soil was prepared by adding phenanthrene to the soil, with a final phenanthrene concentration of 200 mg/kg. The contaminated soil was aged for 1 week.

### 2.3. Activated Sludge Domestication

In this experiment, the activated sludge in the aerobic tank of an urban sewage treatment plant was used as the bacterial source. An inorganic salt medium comprised MgSO_4_•7H_2_O (0.40 g/L), FeSO_4_•7H_2_O (0.002 g/L), K_2_HPO_4_ (0.20 g/L), (NH4)_2_SO_4_ (0.20 g/L), and CaSO_4_ (0.08 g/L). We took 10 mL of activated sludge and added it to 90 mL inorganic salt medium with phenanthrene as the only carbon source. We set the temperature at 30 °C and the shaking rate at 180 rpm, and culture in a constant temperature oscillation box. We increased the concentration of phenanthrene in the culture medium step by step, which is 50 mg/L, 100 mg/L, and 200 mg/L in turn. Finally, the efficient phenanthrene degrading bacteria group was obtained and named MZJ_21. The specific microbial community composition is shown in Figure S1. After enrichment, when the concentration of bacterial solution was more than 1 × 10^8^ CFU/mL, we put it into the aerobic slurry bioreactor at a concentration of 10%, and changed the temperature, aeration rate, and mixing speed of the aerobic slurry bioreactor to find the optimum conditions for degradation of phenanthrene. Finally, the optimal conditions of the aerobic slurry bioreactor were determined as follows: the temperature is 30 °C, the aeration rate is 1 L/min, and the stirring speed is 120 rpm.

### 2.4. Reactor Configuration and Operation

**Figure 1:**
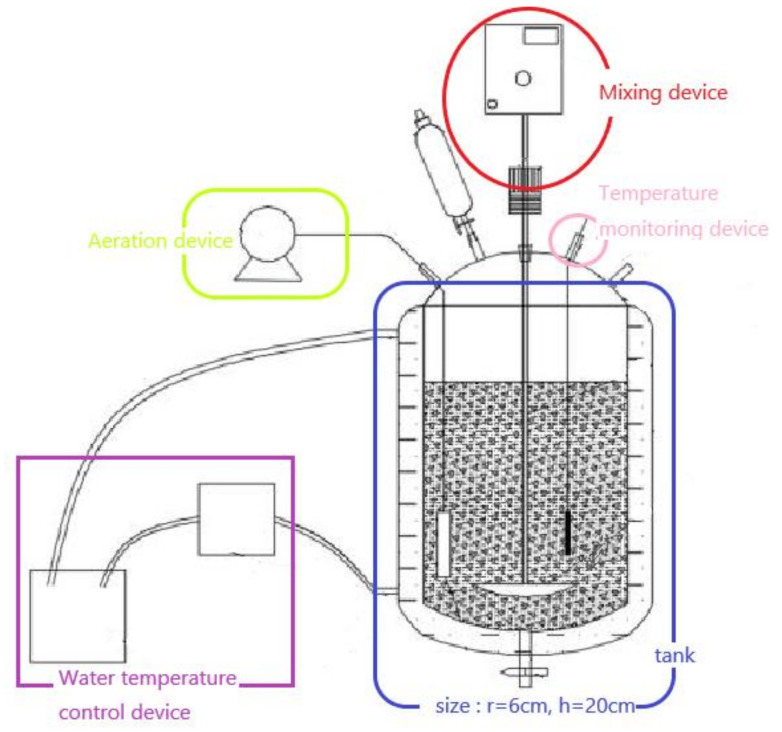
Aerobic slurry bioreactor in the test.

The aerobic slurry bioreactor used in this study is shown in Figure 1. The reactor is constructed from a plexiglass cylinder with the effective volume of 1 L. The aerobic slurry reactor has agitation, aeration, and temperature control. A sampling port is situated at the bottom of the reactor. The reaction solvent, soil, and bacterial solution artificially added in the tank are collectively referred to as mud system. The temperature, stirring speed, and aeration rate were maintained at 30 °C, 120 rpm, and 1 L/min, respectively. The MZJ_21 was used as the microbial inoculation source. 

In order to further explore the reasons for the efficient degradation of phenanthrene by MZJ_21. Three parallel controls were set for each experimental group below, that is, each group of experiments were carried out simultaneously in three aerobic slurry bioreactor. The following comparative experiments were set up:

(1) Natural soil + MZJ_21: Firstly, we accurately weighed 200 g of natural soil with phenanthrene concentration of 200 mg/kg and added it to the aerobic slurry bioreactor. Secondly, we accurately measured 360 mL of inorganic salt medium and 40 mL MZJ_21 bacteria liquid, mixed them evenly, and put the mixture into the aerobic slurry bioreactor. The water–soil ratio in the aerobic slurry bioreactor was 2:1.
(2) Natural soil: Firstly, we accurately weighed 200 g of natural soil with phenanthrene concentration of 200 mg/kg and added it to the aerobic slurry bioreactor. Secondly, we accurately measured 400 mL of inorganic salt medium and put it into the aerobic slurry bioreactor. The water–soil ratio in the aerobic slurry bioreactor was 2:1.
(3) Sterile soil: Firstly, the soil samples used for the tests were sterilized at 121 °C for 30 min. Secondly, we accurately weighed 200 g of sterile soil with phenanthrene concentration of 200 mg/kg and added it to the aerobic slurry bioreactor. The phenanthrene content and soil–water ratio were the same as those in step (2).

After 48 h, the phenanthrene content in the soil was determined. Experiments were repeated three times for each sample group.

### 2.5. Extraction Method of Phenanthrene

As mentioned in the introduction, the solid-liquid mass transfer of phenanthrene in aerobic slurry bioreactor is an important mechanism for studying soil-slurry bioreactors, which greatly affects the bioavailability of phenanthrene. In order to specify the distribution ratio of phenanthrene in solid and liquid phases in aerobic slurry bioreactor, it is necessary to extract the residual phenanthrene in liquid phase and solid phase, respectively. 

#### 2.5.1. Solid Phase

The extraction of phenanthrene from the soil phase was based on EPA 3550C. Phenanthrene was extracted using a mixed extractant (acetone:n-hexane = 1:1) by employing an ultrasonicator (KQ-200VED, Kunshan, Shanghai). Solvent layers were centrifuged at 5000 rpm for 10 min. After nitrogen evaporation, extracted phenanthrene was dissolved in 2 mL of ethyl acetate, and this solution was stored at 4 °C for further analysis.

#### 2.5.2. Liquid Phase

We took 10 mL of evenly mixed mud sample, placed it in a 50 mL centrifuge tube, let it stand for 10 min, collected 1 mL of supernatant and injected it into a 2 mL centrifuge tube, and then added 1 mL ethyl acetate into a 2 mL centrifuge tube. After shaking with a vortex mixer for 20 min, we centrifuged it at 5000 rpm for 10 min. We used a disposable syringe to collect the upper organic phase and pass it through a 0.22 μm organic phase filter membrane, after which we injected it into a GCMS injection bottle and waited for the machine measurement.

### 2.6. Extraction Methods of EPS

In order to explore the reasons for the difference in the distribution of phenanthrene in the solid and liquid phases under different microbial conditions, EPS in different systems were extracted after the reaction. Moreover, cell and EPS of MZJ_21 were isolated to explore the effect of each fraction on phenanthrene allocation.

We took 5 mL of evenly mixed mud in the aerobic slurry bioreactor; the sample was firstly subject to ultrasonication at 40 W in an ice bath for 2 min. The sonicated slurry was then centrifuged at 20,000 r/min, 4 °C for 20 min. The supernatants were filtered through a 0.22 μm membrane. The filtrate was used as the EPS sample. At the same time, we took 40 mL of MZJ_21-enriched communities with a concentration of 1 × 10^8^ CFU/mL, and the extraction method of EPS was the same as the above operation [28]. The supernatants were filtered through a 0.22 μm membrane. The filtrate was used as the EPS. The remaining solid part, resuspended with inorganic salt medium, was used as the cell.

The proportion of polysaccharide and protein in EPS is 70–90%. Therefore, the sum of the two quantities can roughly reflect the total amount of EPS in this experiment. The Lowry Protein Assay Kit (Sangon Biotech Co., Ltd., Shanghai, China) was used to determine the protein concentrations of EPS. The carbohydrate content of EPS was measured using the anthrone–sulphuric acid colorimetric assay. The measurement results are shown in Table S1.

### 2.7. Analysis Methods

#### 2.7.1. Determination of Phenanthrene

Gas chromatography–mass spectrometry (GC-MS) (Clarus SQ 8, Perkin Elmer, Waltham, MA, USA) equipped with a capillary column (30 m × 0.25 mm × 0.25 μm) was used to analyze the phenanthrene concentration. The following GC-MS parameters were used [29]: the ionization mode was electron bombardment (EI), with an ionization energy of 70 eV. The temperatures of the ion source and inlet were 230 and 250 °C, respectively. The temperature increase commenced at 80 °C, and this temperature was maintained for 2 min. Subsequently, the temperature was increased to 200 and 230 °C at a rate of 15 and 4 °C/min, respectively. Finally, the temperature was increased to 280 °C at 10 °C/min; this temperature was maintained for 2 min. The injection method was split injection (1 μL), and the carrier gas was helium (He), introduced at a flow rate of 2 mL/min.

#### 2.7.2. Fluorescent Component of EPS

Three-dimensional excitation emission matrix (3DEEM) fluorescence spectroscopy provides the spectra of fluorescence intensity changes with excitation (Ex) and emission (Em) wavelength changes simultaneously [30]. It can identify and characterize a multi-component complex system of overlapping objects in fluorescence spectra with high selectivity and high information content, and without destroying the sample structure, it is suitable for identifying the components of complex biological macromolecular organic compounds such as extracellular polymers [31].

The fluorescence spectrum of EPS in samples were recorded by a synchronous absorption fluorescence spectrometer (Aqualog-UV-NIR 800C, HORIBA Instruments Incorporated, Piscataway, NJ, USA) [32]. The spectra were measured in 10 mm quartz cuvette, at excitation and emission wavelengths of 200–500 and 200–600 nm, respectively, with 5-nm increments. Ultrapure water was used as the blank during sample analyses. Origin 9.0 was employed for EEM data processing. 

#### 2.7.3. Microbial Diversity Analysis

The bacterial consortium before and after acclimation and soil slurry acquired from the reaction with the natural soil or natural soil + consortium were sampled and Shanghai Majorbio Bio-pharm Technology Co., Ltd., Shanghai, China. was entrusted to detect the bacterial community. Using microbial genomic DNA as template, the v3–v4 variable region of 16S rRNA gene was amplified with primers 515F (5′-gtgccagcgggcgcgg-3′) and 907R (5′-ccgtcaattcmtragtt-3′). The 50 μL PCR amplification system: 5 × Primerstar buffer 10 μL, dNTPs 2 μL, 515F 2μL, 907R 2 μL, DNA template 2 μL, primerstar polymerase 1 μL, sterile ddH_2_O 31 μL. PCR amplification procedure: denaturation at 95 °C for 10 min, denaturation at 95 °C for 30 s, annealing at 52 °C for 30 s, extension at 72 °C for 30 s, 30 cycles, extension at 72 °C for 10 min; electrophoresis detection amplification results. The amplified products were detected by 2% agarose gel electrophoresis, and the products of polymerase chain reaction (PCR) were quantified by TBS-380 (Turner Biosystems, Sunnyvale, CA, USA). Then, the DNA library was constructed and run on the Illumina miseq platform of Shanghai Majorbio Bio-pharm Technology Co., Ltd., Shanghai, China. The 97% similarity threshold is used to aggregate high-quality non chimeric sequences into the operational classification unit (OTU) and the uclust algorithm, with the best uclust setting being implemented in qiime.

#### 2.7.4. Analysis of Other Indicators

Scanning electron microscopy (SEM, FEI, USA) was used to analyze the bacterial distribution in the soil phase. 

Colony-forming units (CFUs) were used to determine the number of bacteria in the soil and liquid phase. The specific operation is as follows:

Firstly, 10 mL of slurry sample was poured into the centrifuge tube, centrifuged at 5000 rpm for 10 min, the supernatant was collected and diluted 10 times, and then the liquid was used as the bacterial solution sample in water. Secondly, 10 mL deionized water was add into the centrifuge tube containing only soil, shaken and mixed manually, extracted by ultrasonic for 30 min, and centrifuged at 5000 rpm for 10 min, after which the supernatant was absorb and diluted 10 times. This liquid is used as the sample of bacterial solution in soil.

Solid medium: NH_4_Cl 2.0 g, KH _2_PO_4_ 1.5 g, Na_2_HPO_4_ 0.5 g, CaCl_2_ 0.01 g, MgCl_2_ 0.2 g, agar 15.0–20.0 g, deionized water 1 L, pH = 7. The solid medium was sterilized at 121 °C for 30 min. After cooling to about 45 °C, 15–20 mL solid medium was poured into a sterile petri dish. After the agar was cooled and solidified, 0.2 mL of diluted bacterial solution was collected and applied to the plate. This was incubated in a 30 °C incubator for 7 days. When colonies appear, they were counted directly under the microscope with a blood cell counting plate.

## 3. Results

### 3.1. Degradation of Phenanthrene in Aerobic Slurry Bioreactor

**Figure 2:**
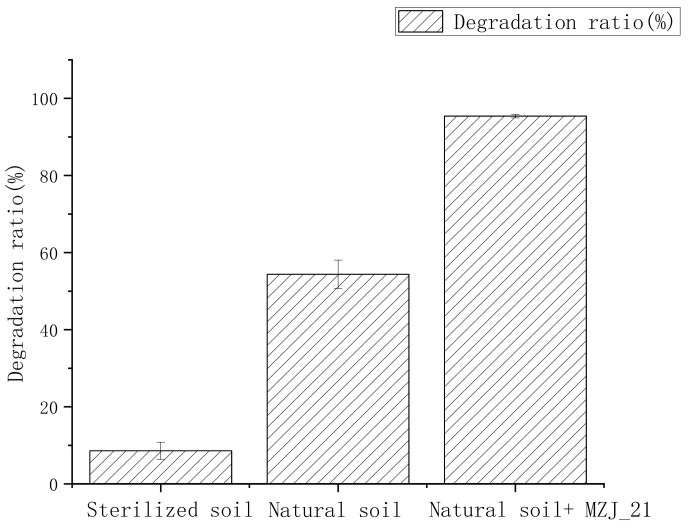
Degradation of phenanthrene in the slurry reactor.

Figure 2 shows the degradation rate of phenanthrene in the sterilized soil, natural soil, and natural soil with MZJ_21.

After 48 h, only 8.6% phenanthrene was removed in the sterilized soil, and in natural soil, the phenanthrene degradation efficiency was 54.38%. The phenanthrene degradation rate in the natural soil reactor with MZJ_21 was 95.41%, which was 1.75 times higher than that without MZJ_21. The addition of MZJ_21 communities in the slurry reactor substantially improved the phenanthrene degradation rate. It can be seen that the difference of microorganisms affects the degradation rate of phenanthrene, so we explored the situation of microorganisms in the system. Firstly, we count the number of microorganisms in the solid phase and liquid phase by plate culture method. The experimental error comes from the error between three parallel samples.

The results showed that the density of the culturable bacteria in sterilized soil, natural soil, and natural soil plus bacteria are about 5.67 × 10^3^, 1.14 × 10^7^, and 6.31 × 10^7^, respectively (Table 1). In the process of aeration, microorganisms in the air will be introduced into the aerobic slurry bioreactor. Microorganisms in natural soil can also grow in biological mud reactor. Meanwhile, MZJ_21 can well adapt to the environmental conditions of aerobic slurry bioreactor. The number of microorganisms in natural soil + MZJ_21 is 5.6 times higher than in natural soil. This indicates that the interface of microbial degradation of phenanthrene in aerobic slurry bioreactor may be in the solid phase. 

### 3.2. Distribution of Phenanthrene in the Aerobic Slurry Bioreactor

**Figure 3:**
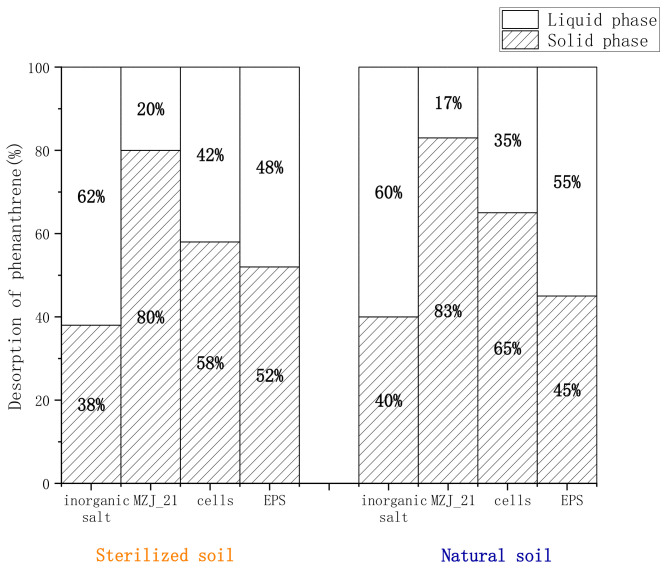
Distribution proportion of phenanthrene in the liquid and solid phases.

We isolated bacterial cell and extracellular polymers to explore the specific mechanism of the MZJ_21 on the distribution of phenanthrene in the system. Since MZJ_21 communities, cells, and EPS all use the inorganic salt culture medium as the solvent, the inorganic salt culture medium is set as the blank control. In addition, in order to eliminate the influence caused by the change of phenanthrene degradation, the duration of this experiment is set to 1 h. It can be seen from Figure 3 that there is no significant difference in the distribution proportion of phenanthrene in solid phase and liquid phase between sterilized soil and natural soil. It shows that the existence of original microorganisms in natural soil has little effect on the distribution of phenanthrene by MZJ_21 communities, cells, and EPS. Taking the natural soil group as an example, the distribution proportion of phenanthrene in the solid phase in the experimental group supplemented with MZJ_21 communities, cells, and EPS was 83%, 65%, and 55%, respectively. Compared with the group supplemented with inorganic salt medium (40%), it increased by 1.08 times, 0.63 times, and 0.38 times, respectively. Obviously, microbial components (MZJ_21 communities, cells, EPS) improve the distribution ratio of phenanthrene in the solid phase. Among them, the reason for the increase of cell distribution ratio may be the active adsorption of microorganisms. It is worth noting that the addition of EPS alone in the system also significantly improves the distribution of phenanthrene in the solid phase, which may be why EPS with electronegativity and a large number of functional groups is easy to bind to soil particles, and adsorbs phenanthrene in the liquid phase to provide reaction sites for the degradation of phenanthrene. When cells and EPS exist together, they play a greater role than when they exist alone.

Moreover, it can be seen from Figure 3 that after adding the inorganic salt, the content of phenanthrene in the liquid phase is relatively large, which is caused by the fact that phenanthrene still exists in the organic phase in the liquid phase. The phenanthrene is a lipophilic organic substance and is insoluble in water, so we use methanol to dissolve the phenanthrene to prepare a phenanthrene-containing methanol solution. However, the content of phenanthrene in the liquid phase of the added MZJ_21, cells, and EPS was less, indicating that the phenanthrene had been transferred from the liquid phase to the solid phase through mass transfer. This also shows that microorganisms can promote the transfer of phenanthrene from the liquid phase to the solid phase, thereby improving the degradation efficiency of phenanthrene by microorganisms.

### 3.3. Effect of Extracellular Polymers on the Degradation Rate of Phenanthrene

**Figure 4:**
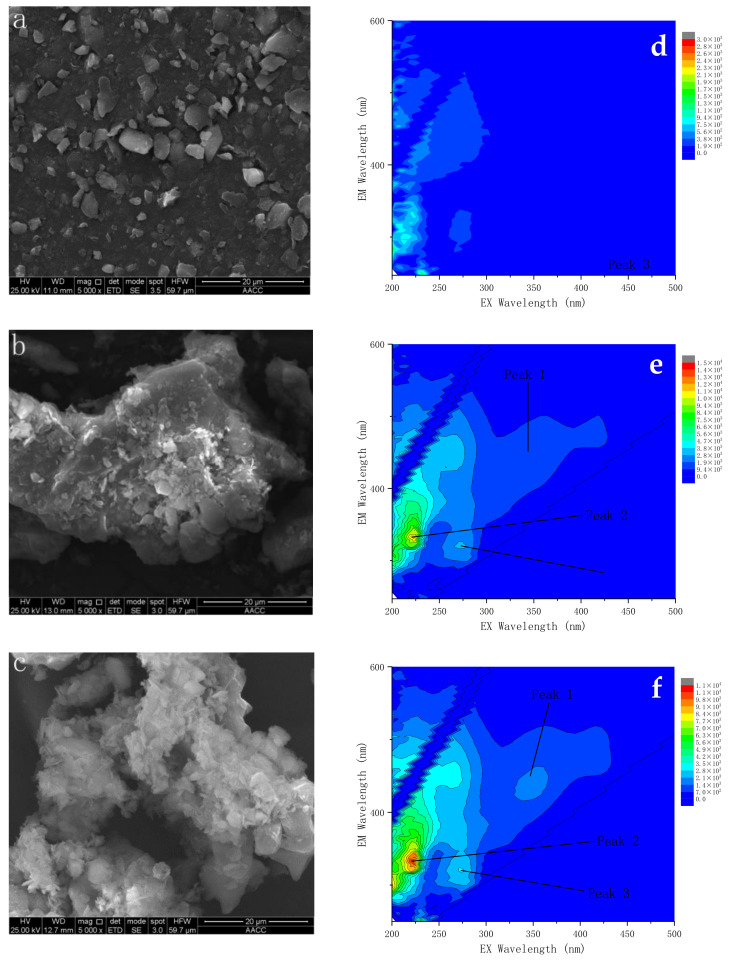
SEM images of (**a**) sterilized soil, (**b**) natural soil, and (**c**) natural soil + MZJ_21. Three-dimensional fluorescence monitoring of TB-EPS in consortium and mud systems: (**d**) natural soil, (**e**) consortium, and (**f**) natural soil + MZJ_21.3.4. Diversity of the Bacterial Community.

The SEM images (Figure 4a–c) show that the surface of the sterilized soil is smooth, and no bacteria adhere to this soil. However, many bacteria attached to the surface of natural soils and natural soils with MZJ_21. To further explore the cause for MZJ_21 to well adhere to the soil particle surface, 3D fluorescence analysis was performed for the three groups. Figure 4d–f shows the fluorescence peaks of excitation/emission wavelength (Ex/Em). The peak intensity presented in Figure 4f is the highest, which indicates that the EPS content of the natural soil with MZJ_21 is considerably higher than that of the other two groups. It can be seen that the amount of EPS produced by microorganisms in aerobic slurry bioreactor increases after the addition of communities, especially in Peaks 1, 2, and 3. Through 3D-EEM, in EPS of the bacterial consortium, two chemical components, namely proteins and tryptophan, were identified. The specified proteins included aromatic proteins (peak 1; Ex/Em 220/325–335) [33].

Based on the number of OTUs, Table 2 lists the microbial diversity indexes in reactor added with the MZJ_21, natural soil, and natural soil + MZJ_21 after 48 h of reaction. The microbial diversity indicators include sobs, Shannon, Simpson, ACE, Chao, and coverage. The value of the sobs index is directly related to the number of OTUs obtained by sequencing. Shannon and Simpson index jointly reflect the species diversity of the sample, while ACE and Chao index jointly reflect the abundance of the sample species. The sequencing results show a high coverage of OTUs in the three samples, which is higher than 98.9%. This means that the results of microbial diversity can reflect the real composition of microorganisms in the samples. We found that compared with microorganisms in natural soil, MZJ_21 has lower species diversity and abundance, which is undoubtedly the result of domestication. Combined with the experiment of plate culturable bacteria in 2.8.4, we infer that the microorganisms that can utilize organic matter and highly degrade phenanthrene account for only a small part of all microorganisms, but they play a great role in degradation function.

**Figure 5:**
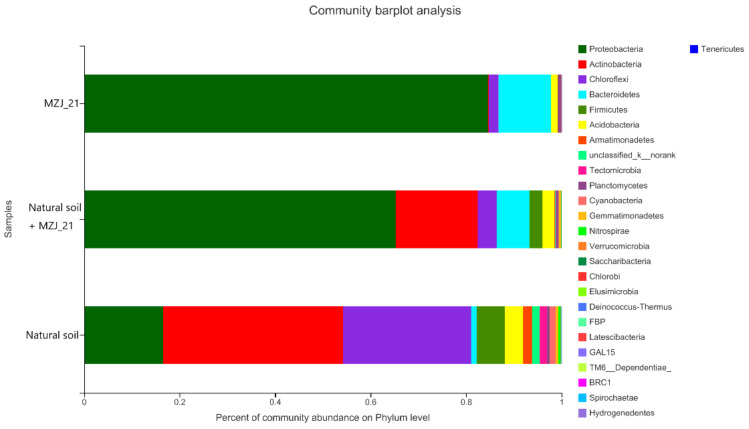
Phylum-level community distribution of bacteria in different experimental groups.

Community analysis at the phylum level generally (Figure 5) shows the composition differences of microorganisms in MZJ_21, MZJ_21 + Natural Soil, and Natural Soil. In the MZJ_21 consortium, *Proteobacteria* (84.64%), *Bacteroidetes* (10.97%), and *Chloroflexi* (2.03%) accounted for over 95% of the bacterial community. The composition of the natural soil community is more complex than that of the domesticated communities, which can reflect the microbial composition of ordinary soil. *Actinobacteria* (37.70%), *Chloroflexi* (26.73%), and *Proteobacteria* (16.37%) are the most abundant phylum in this community, followed by *Firmicutes* (5.84%), *Acidobaceria* (3.87%), *Armatimonadetes* (1.85%), *Tectomicrobia* (1.54%), *Cyano-bacteria* (1.29%), and others. The microbial community composition of MZJ_21 + Natural Soil is similar to that of Natural Soil, but the proportion of *Proteobacteria* increased, and the proportion of photosynthetic autotrophic bacterium *Chloroflexi* and aerobic saprophytic bacterium *Actinobacteria* decreased. *Proteobacteria* (65.23%), *Actinobacteria* (17%), and *Bacteroidetes* (7.03%) became the most abundant phylum in the sample, followed by *Chloroflexi* (3.97%), *Firmicutes* (2.69%), and *Acidobacteria* (2.52%).

**Figure 6:**
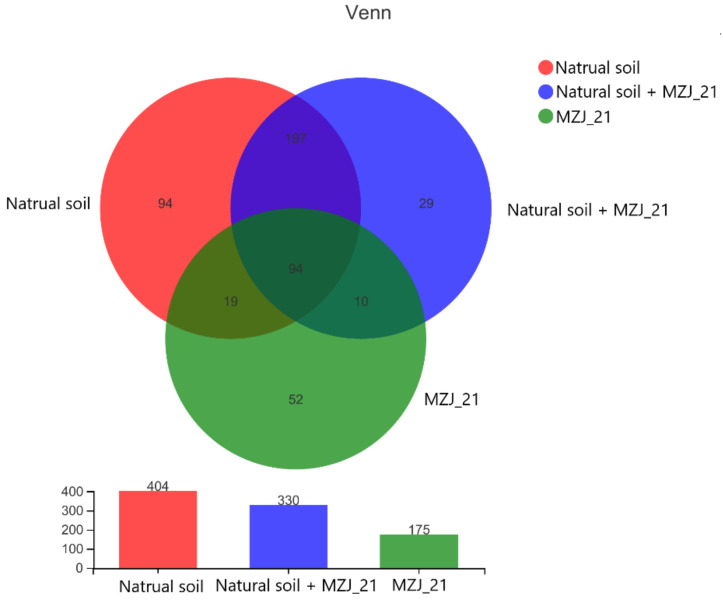
Venn diagram at genus level for different experimental groups.

Combined with a Venn diagram at genus level (Figure 6), we obtained the following information: there are 197 common genera of communities in Natural soil and Natural soil + MZJ_21, which may contain bacteria that can degrade organics, such as phenanthrene in the natural environment; MZJ_21 and natural soil + MZJ_21 share 10 unique genera. They might be the important genus with functional capacity that can play an effective role in reducing phenanthrene. There are 29 endemic genera in Natural soil + MZJ_21, and the most likely source is the new adaptive genus that evolved after MZJ_21 was mixed with natural soil.

**Figure 7:**
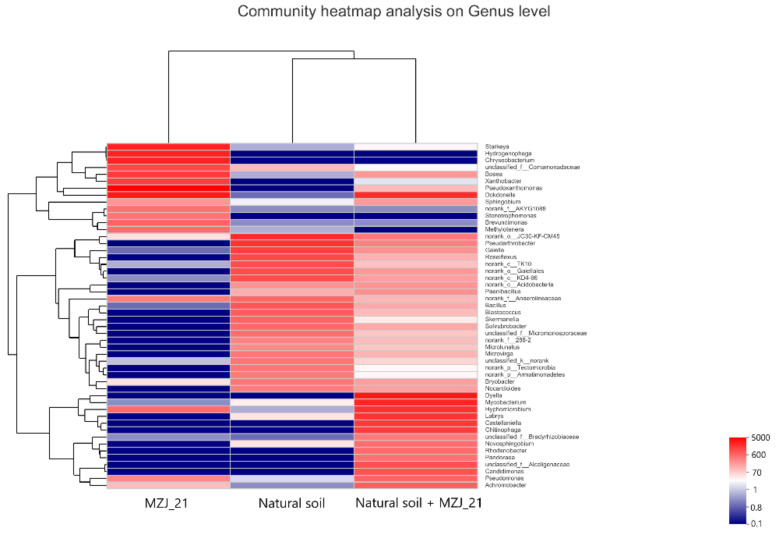
Genus thermogram and clustering tree for different experimental groups.

The heat map at the genus level (Figure 7) reflects the differences of microorganisms that have the main functions in the three samples in more detail. The dominant genus in MZJ_21 are represented by *Pseudoxanthomonas* (19.29%), *Dokdonella* (11.12%), *Hydrogenophaga* (9.36%), *Starkeya* (9.30%), *Chryseobacterium* (9.30%), and *Bosea* (5.65%). *Pseudoxanthomonas, Dokdonella*, and *Starkeya* are aerobic bacteria and can play an important role in the degradation of polycyclic aromatic hydrocarbons [34,35,36]. In addition, *Hydrogenophaga* is a facultative hydrogen-loving autotrophic bacterium, which can degrade high molecular weight polycyclic aromatic hydrocarbons in both aerobic and anaerobic environments. In aerobic environment, the degradation ratio of pyrene by hydrogenophaga can reach 94% within 15 days [37]. The dominant genera of natural soil communities include *Pseudarthrobacter* *Gaiella*, *Roseiflexus*, and some *noranked genera JG30-KF-CM45*, *TK10no*, *Gaiellales*, and *KD4-96*. When the two are mixed and reacted in an aerobic bioreactor for a period of time, a series of genera with no significant advantages in communities and natural soil appear in the system, such as *Dyella*, *Mycobacterium*, *Hypomicrobium*, *Labrys*, *Castellaniella*, *Chitinophaga*, etc.

## 4. Discussion

MZJ_21 domesticated in this experiment is a microbial community with high phenanthrene degradation efficiency. Compared with many individual strains, MZJ_21 shows higher degradation efficiency and greater advantages. For example, Chaudhary et al. isolated a bacterial strain named *Streptomyces rochei PAH-13*, the isolate was found to degrade phenanthrene between 80–85% within 15 days at 100 ppm level [38]. Zhong et al. isolated pure single strain of *Bordetella petrii* from polluted soil. The degradation ability of *Bordetella petrii* to phenanthrene is not very stable, and the degradation ratio is only 80% within 7 days at 100 ppm level [39]. At present, there is no specific model strain for phenanthrene degradation. This is because the isolation and purification process of single strain is time-consuming and costly, and it is easy to introduce other bacteria even after purification. In addition, the adaptability of single strain to the environment is poor. Moreover, as shown in this experiment, microorganisms in the natural environment also have considerable phenanthrene degradation ability, so strict pure culture of strains is not necessary. After simple domestication, MZJ_21, as a cooperative group of multiple bacteria, shows stronger environmental adaptability and population structure and functional stability. This provides an idea for the selection of microorganisms in biological mud reactor. 

**Figure 8:**
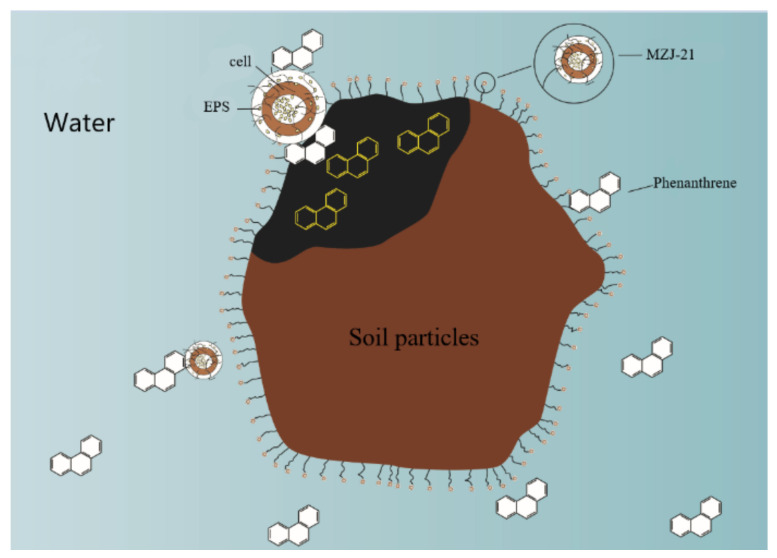
Strategy of phenanthrene degradation by MZJ_21 in solid phase.

The results of agar plate culture experiment (Table 1) can reflect that there are more microorganisms with available organic matter in the solid, and MZJ_21 is easier to exist in the solid phase; the results of electron microscope experiments also verify this phenomenon. Simultaneously, the experimental results of phenanthrene distribution in solid and liquid phases with the participation of microorganisms show that most phenanthrene exists in the solid phase, which is closely related to the role of MZJ_21. Based on the above experimental results, the main mechanism of phenanthrene degradation by bacteria in biological mud reactor can be inferred (Figure 8). First, MZJ_21 can produce a large amount of EPS, which can affect the ability of microorganisms to contact pollutants. Meanwhile, microbial EPS has many biological functions, including adsorption, promoting hydrophobic substrate uptake and assisting cell adhesion [40,41]. Therefore, MZJ_21 can be actively adsorbed on soil particles through EPS assisted adhesion and bacteria, and the soil particles become a place for MZJ_21 to reduce phenanthrene. Secondly, MZJ_21 can also use the adsorption of EPS to adsorb phenanthrene in liquid phase and solid phase, improve the probability of microorganism exposure to phenanthrene, so as to strengthen the degradation ability of MZJ_21 to phenanthrene.

In conclusion, the highly efficient phenanthrene degrading bacteria in MZJ_21 exist in the solid phase, so it can be inferred that the process of phenanthrene degradation by MZJ_21 may occur in the solid phase. Chunyun et al. [26] investigate the differential contribution of extracellular polymeric substances (EPS) to polycyclic aromatic hydrocarbon (PAH) degradation. It was found that the biodegradation of pyrene and B[a] P increased after EPS were introduced into the PAH degradation solution, and the higher the protein and carbohydrate content of EPS, the higher the ability of EPS to degrade polycyclic aromatic hydrocarbons. Studies have shown that differential bacterial growth, enzymatic activity, and EPS composition may affect the biodegradation of polycyclic aromatic hydrocarbons [42]. The hypothesis that extracellular polymeric substances (EPS) affect the formation of biofilms for subsequent enhanced biodegradation of polycyclic aromatic hydrocarbons was tested. Moreover, Zhang propose that the bacterial-produced EPS was a key factor to mediate bacterial attachment to other surfaces and develop biofilms, thereby increasing the bioavailability of poorly soluble PAH for enhanced biodegradation [27].

## 5. Conclusions

Biota MZJ_21 with high phenanthrene-degradation efficiency was domesticated in this experiment. The dominant genus in MZJ_21 is represented by *Pseudoxanthomonas* (19.29%), *Dokdonella* (11.12%), *Starkeya* (9.36%), and *Hydrogenophaga* (9.30%). The experimental results showed that after the addition of MZJ_21 to the aerobic slurry bioreactor, with the optimum conditions of temperature, stirring speed, and aeration rate of 30 °C, 120 rpm, and 1 L/min, respectively, the phenanthrene degradation ratio reached 95.41% within 48 h. Compared with bacteria in natural soil, the survival ability of MZJ_21 in solid phase is stronger, which can reach 6.25 ± 0.36 × 10^7^ CFU/g. It is found that MZJ_21 can produce more EPS, which may enhance the distribution proportion of phenanthrene in the solid phase. It is found that MZJ_21, cells, and EPS can improve the distribution proportion of phenanthrene in the solid phase. From this, we conclude that MZJ_21 can effectively degrade phenanthrene in the solid phase through active adsorption and the auxiliary adhesion of EPS.

## Figures and Tables

**Table 1:** Number of bacteria in the soil and water of the slurry reactor.

	Sterilized Soil	Natural Soil	Natural Soil + MZI_21
Liquid phase (CFU/mL)	3.65 ± 0.04 × 10^3^	3.78 ± 0.16 × 10^5^	6.45 ± 0.23 × 10^5^
Solid phase (CFU/g)	2.02 ± 0.07 × 10^3^	1.11 ± 0.33 × 10^7^	6.25 ± 0.36 × 10^7^

**Table 2:** Alpha diversity index.

Sample	Sobs	Shannon	Simpson	ACE	Chao	Coverage
MZJ_21	377	3.159	0.0849	489.159	484.018	0.997
Natural Soil	1493	5.697	0.0122	1633.885	1643.707	0.994
MZJ_21 + Natural Soil	949	4.1001	0.0415	1318.331	1362.255	0.989

## Data Availability

The data presented in this study are available on request from the corresponding or first author.

## Supplementary Materials

The following supporting information can be downloaded at: https://www.mdpi.com/article/10.3390/ijerph19095515/s1, Figure S1: Community analysis pieplot on Genus level: MZJ_21; Table S1: Contents of EPS polysaccharide and protein.
