# Supplementary material for: Strategy to Promote the Biodegradation of Phenanthrene in Contaminated Soil by a Novel Bacterial Consortium in Slurry Bioreactors
Source: Int J Environ Res Public Health. 2022 May 1;19(9):5515. doi: 10.3390/ijerph19095515 (PMC9101024; doi:10.3390/ijerph19095515)
Supplement: Supplementary file 1 [file ijerph-19-05515-s001.zip › ijerph-1647278-supplementary.pdf]

International Journal of
Environmental Research and Public Health

MDPI

Table S1. Contents of EPS polysaccharide and protein.

**Sample**	protein	polysaccharide
MZJ_21	108.8	61.50
Natural Soil	43.80	7.13
Natural Soil + MZJ_21	117.80	44.625
Sterilized Soil	10.21	3.29

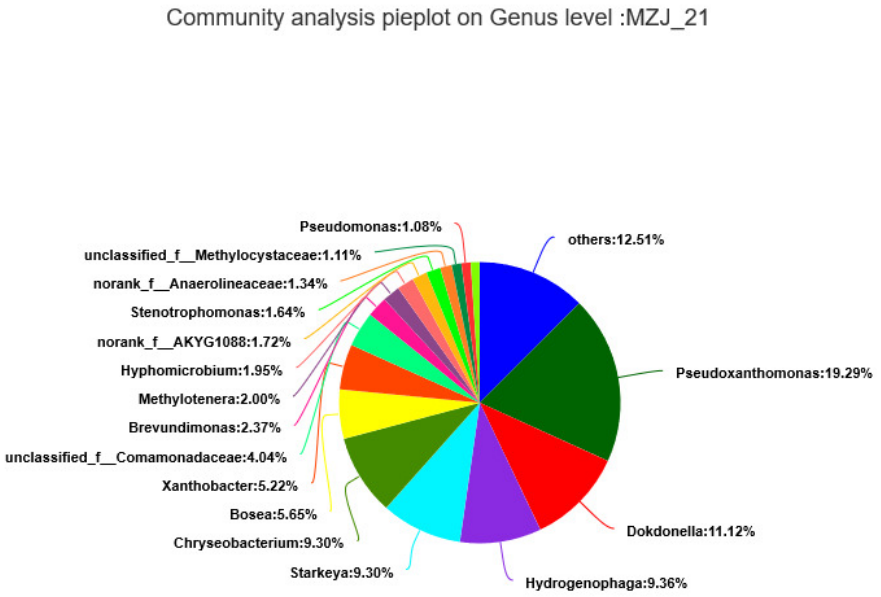

Figure S1. Community analysis pieplot on Genus level: MZJ_21.
